# Neuroticism Trait and Mental Health Among Chinese Firefighters: The Moderating Role of Perceived Organizational Support and the Mediating Role of Burnout—A Path Analysis

**DOI:** 10.3389/fpubh.2022.870772

**Published:** 2022-04-05

**Authors:** Yanqiang Tao, Zijuan Ma, Wenxin Hou, Yuanyuan Zhu, Liang Zhang, Chunbo Li, Congying Shi

**Affiliations:** ^1^Beijing Key Laboratory of Applied Experimental Psychology, School of Psychology, Beijing Normal University, Beijing, China; ^2^School of Psychology, South China Normal University, Guangzhou, China; ^3^School of Health Professions Education, Maastricht University, Maastricht, Netherlands; ^4^Student Mental Health Education Center, Northeast Agricultural University, Harbin, China; ^5^College of Education for the Future, Beijing Normal University, Zhuhai, China; ^6^School of Psychology, Nanjing Normal University, Nanjing, China

**Keywords:** neuroticism, job burnout, perceived organization support, anxiety, depression

## Abstract

Perceived organizational support (POS) in the relationship between neuroticism and job burnout among firefighters received little attention in China. A sampling of 716 firefighters in China, we drew on perceived organization support theory and the notion of support as a buffer in job burnout, examining moderating effects of POS on the relationship between neuroticism and three components of burnout (emotional exhaustion, depersonalization and reduced personal accomplishment). Also, this study explored the mediating effect of burnout on the relationship between neuroticism and mental health (i.e., anxiety and depression). We found that two components (depersonalization and emotional exhaustion) of burnout have significantly mediated the relationship between neuroticism and anxiety and depression. At the same time, POS reinforced the relationship between neuroticism and depersonalization and emotional exhaustion. Therefore, organizations can take our analysis into account when taking actions to improve firefighters' mental health. The implications of these findings were discussed.

## Introduction

The Chinese government faces the challenge of an increasing number of disasters and accidents along with its rapid economic and social development. As a result, Chinese firefighters need to tackle increasing fires and respond to other emergencies, resulting in a high death rate (1). Firefighters are working as first responders who face emergent tasks, including fire suppression and rescue services which may cause severe injuries or deaths. Witnessing long-lasting life-threatening events and tragedies occur on colleagues can negatively affect a person's mental and physical health (2), causing anxiety (3, 4) and depression (5, 6). Moreover, without enough external assistance and organizational support, firefighters' mental problems become a more challenging issue that needs to be tackled urgently.

The overarching idea of our study was to find the impact of organizational support and how personality traits, especially neuroticism, influences firefighters' mental health. Neuroticism is a negative emotional trait characterized by proneness to anxiety, emotional instability, and self-consciousness (7). The personality trait of neuroticism is associated with anxiety and depression (8–12). A body of research has identified neuroticism as a vulnerable factor in both depressions (13, 14) and anxiety (15, 16).

### The Moderating Role of Perceived Organizational Support

Perceived Organizational Support (POS) is defined as employees' perception about how their organization values their contribution and cares about their wellbeing (17). A previous study found that POS was negatively correlated with job burnout of employees, meaning that the less POS, the fewer satisfaction employees would feel, resulting in more severe burnout (18). According to Cohen and Wills (19), support can promote personal self-esteem and provide sufficient information to individuals to help them define, understand, and respond to stressful events. Meanwhile, support has a function to provide physical resources and has a social companionship function to satisfy people's need to be accompanied and feel a sense of belonging (20). Organizational support is especially meaningful for researching burnout, which has long been recognized as a combination of work-related symptoms, including generalized fatigue and loss of motivation (21). Although burnout is linked to general personality factors (22), there has been little research on the relationship between firefighters' neuroticism and burnout. Particularly, firefighters who perceive their organizations are supportive will perform better, believing that organizations will provide them with resources to cope with the stress leading to less burnout.

### The Mediating Role of Burnout

Burnout is a psychological syndrome characterized by emotional exhaustion, feelings of cynicism, and reduced personal accomplishment (23). A recent study has shown that job burnout is one of the risk factors for anxiety and depression (24). Different elements of burnout contribute to mental health issues, in which exhaustion was positively correlated with depression, and the sense of professional inefficacy was positively correlated with anxiety (25). Vasilopoulos (26) found that participants who reported a high level of social anxiety also reported a high level of burnout. Mark and Smith (27) revealed that job demands, external efforts, and over-commitment were related to increased anxiety levels. Burnout might be a risky factor for developing depression (28, 29). Therefore, job burnout may be used as a diagnostic standard to assess employees' mental health conditions, such as anxiety and depression. Neuroticism, as a susceptible personality trait, is positively related to anxiety and depression, which means neurotic employees have higher tendency to experience job burnout. Research for firefighters, a particular group for our society, is critical but rare, especially for neurotic firefighters. Hence, our study took neurotic personality into account to establish models to explore how burnout can mediate the relationship between such personality and anxiety or depression.

## Methods

### Participants

The current study recruited 716 full-time male professional firefighters in China who voluntarily participated in this study. The mean age was 26.39 years old, and the average month of work experience is 31.08 within the final data. The ethics committees from all authors' universities approved this study. Informed consent was obtained from participants before they started.

### Measures

#### Big Five Inventory−2

The BFI-2 is a 60-item self-report measure of personality traits (30), and each item is rated on a 5-point Likert scale (1 = strongly disagree; 5 = strongly agree). The BFI-2 consists of five subscales with 12 items each: extraversion, agreeableness, conscientiousness, negative emotionality (neuroticism), and open-mindedness. The higher scores on one trait, the more probable the subject has such trait.

A previous study indicated that the Chinese version of the BFI-2 questionnaire to evaluate personality traits has good reliability and structural validity (31). The current study showed good internal consistency, α = 0.74, 0.84, 0.85, 0.82, 0.76 for extraversion, agreeableness, conscientiousness, negative emotionality (neuroticism) and open-mindedness, respectively.

#### Maslasch Burnout Inventory-General Survey

Maslach and Schaufei's MBI-GS has a good validation across occupational groups and nations (32). The Chinese version (CMBI-GS) was revised by Li Chaoping (33). It consists of 15 items, and all were rated on a 7-point Likert scale (1 = never, 7 = every day). The CMBI-GS composes of three subscales: emotional exhaustion, depersonalization, and reduced personal accomplishment. The higher the score of emotional exhaustion and depersonalization and the lower score in reduced personal achievement, the higher the probability for the subject to have job burnout.

In this study, Cronbach's alpha coefficient for emotional exhaustion, depersonalization, and reduced personal accomplishment were 0.92, 0.93, and 0.91, respectively.

#### Self-Reported Anxiety Scale and Depression Scale

Zung (34) and Zung (35) designed the Self-Rating Anxiety Scale (SAS) and Self-Rating Depression Scale (SDS), respectively, to quantify the degree of anxiety and depression symptoms. These scales both include 20 self-report items (4-point Likert scale). Mean values were calculated. The higher the scores, the higher the inclination for the subject to have anxiety or depression.

The Chinese versions have been validated in epidemiological surveys (36, 37). The current study showed good internal consistency (α = 0.83, 0.85, respectively) for anxiety and depression.

#### Perceived Organizational Support Scale

The perceived organizational support (POS) was measured by using the 8-item scale (38). The short version of the POSS has been widely applied among Chinese occupational groups with good reliability and validity (39).

In the present study, the alpha coefficient for this scale was 0.70.

### Statistical Analysis

In the present study, all analyses were calculated in R 4.1.1 (40). Common method deviation tests, descriptive analyses, and normal distribution were performed using the *psych* package (41). The correlation matrix table was made by using the *apaTables* package (42). We conducted multiple-level mediation analysis by using the *lavaan* package (43) and visualized the pathway by the *lavaanPlot* package (44).

Neuroticism was regarded as an independent variable (IV). Depression and anxiety were set as dependent variables (DV). Job burnout consisted of three components: emotional exhaustion (EE), depersonalization (DE), and reduced personal accomplishment (RPA), and they were regarded as mediation variables (MV). The moderation variable was the perceived organizational support (MDV: POS). Before analysis, we used G^*^Power 3.1 software (45) to calculate the sample size. The result showed that the minimum sample size should be 191 for path analysis with an effect size of 0.3 (46), and the degree of freedom was 3. In this study, we had a sample size of 716 firefighters, which is much more than the critical value.

## Results

### Common Method Deviation Test

The Harman single factor test showed that the eigenvalues of 18 factors were more outstanding than one without rotation, and the explanatory variation of the first factor was 23.56%, lower than the critical value of 40% (47). Therefore, there was no obvious common methodological bias in this study.

### Descriptive Statistics and Correlations Among Main Measures

As Table 1 shows, the neuroticism trait was significantly positive correlated with anxiety (*r* = 0.50, *p* < 0.01) and depression (*r* = 0.29, *p* < 0.01). As expected, firefighters with higher neuroticism, anxiety and depression would experience more burnout (*p* < 0.01). The perceived organizational support was significantly negatively correlated with depression (*r* = 0.08, *p* < 0.05) and neuroticism (*r* = −0.09, *p* < 0.01), while it was not strongly correlated with anxiety (*r* = 0.03, *p* > 0.05). Age was significantly negatively correlated with the neuroticism trait (*r* = −0.10, *p* < 0.05).

**Table 1:** Means, standard deviations, and correlations with confidence intervals for the main study variables.

**Variable**	***M***	***SD***	**1**	**2**	**3**	**4**	**5**	**6**	**7**	**8**
1. Age	26.39	6.00								
2. Work experience	31.08	45.10	0.35^**^ [0.29, 0.41]							
3. SAS	1.86	0.40	−0.07 [−0.14, 0.00]	−0.04 [−0.11, 0.04]						
4. SDS	2.22	0.42	−0.05 [−0.12, 0.02]	−0.09^*^ [−0.16, −0.01]	0.49^**^ [0.43, 0.54]					
5. POS	3.14	0.69	0.03 [−0.04, 0.11]	−0.01 [−0.08, 0.07]	0.03 [−0.04, 0.10]	−0.08^*^ [−0.15, −0.00]				
6. EE	2.76	1.34	−0.09^*^ [−0.16, −0.01]	0.00 [–0.07, 0.07]	0.59^**^ [0.54, 0.63]	0.28^**^ [0.21, 0.34]	0.01 [−0.07, 0.08]			
7. DE	2.29	1.39	−0.10^*^ [−0.17, −0.02]	−0.06 [−0.13, 0.01]	0.62^**^ [0.57, 0.66]	0.28^**^ [0.21, 0.35]	−0.00 [−0.08, 0.07]	0.79^**^ [0.76, 0.81]		
8. RPA	4.66	1.76	0.12^**^ [0.05, 0.19]	0.12^**^ [0.05, 0.19]	−0.26^**^ [−0.33, −0.19]	−0.17^**^ [−0.24, −0.09]	0.12^**^ [0.05, 0.19]	−0.38^**^ [−0.44, −0.32]	−0.41^**^ [−0.47, −0.35]	
9. Neuroticism	2.30	0.68	−0.10^**^ [−0.18, −0.03]	−0.03 [−0.10, 0.04]	0.50^**^ [0.44, 0.55]	0.29^**^ [0.22, 0.36]	−0.09^*^ [−0.17, −0.02]	0.44^**^ [0.38, 0.50]	0.42^**^ [0.36, 0.48]	−0.34^**^ [−0.40, −0.27]

*M and SD are used to represent mean and standard deviation, respectively. Values in square brackets indicate the 95% confidence interval for each correlation. The confidence interval is a plausible range of population correlations that could have caused the sample correlation (48).*

* *Indicates p < 0.05.*

** *Indicates p < 0.01.*

*SAS, Self-Rating Anxiety Scale; SDS, Self-rating depression scale; POS, perceived organizational support; EE, emotional exhaustion; DE, depersonalization; RPA, reduced personal accomplishment*.

### Moderation Analysis

**Figure 1:**
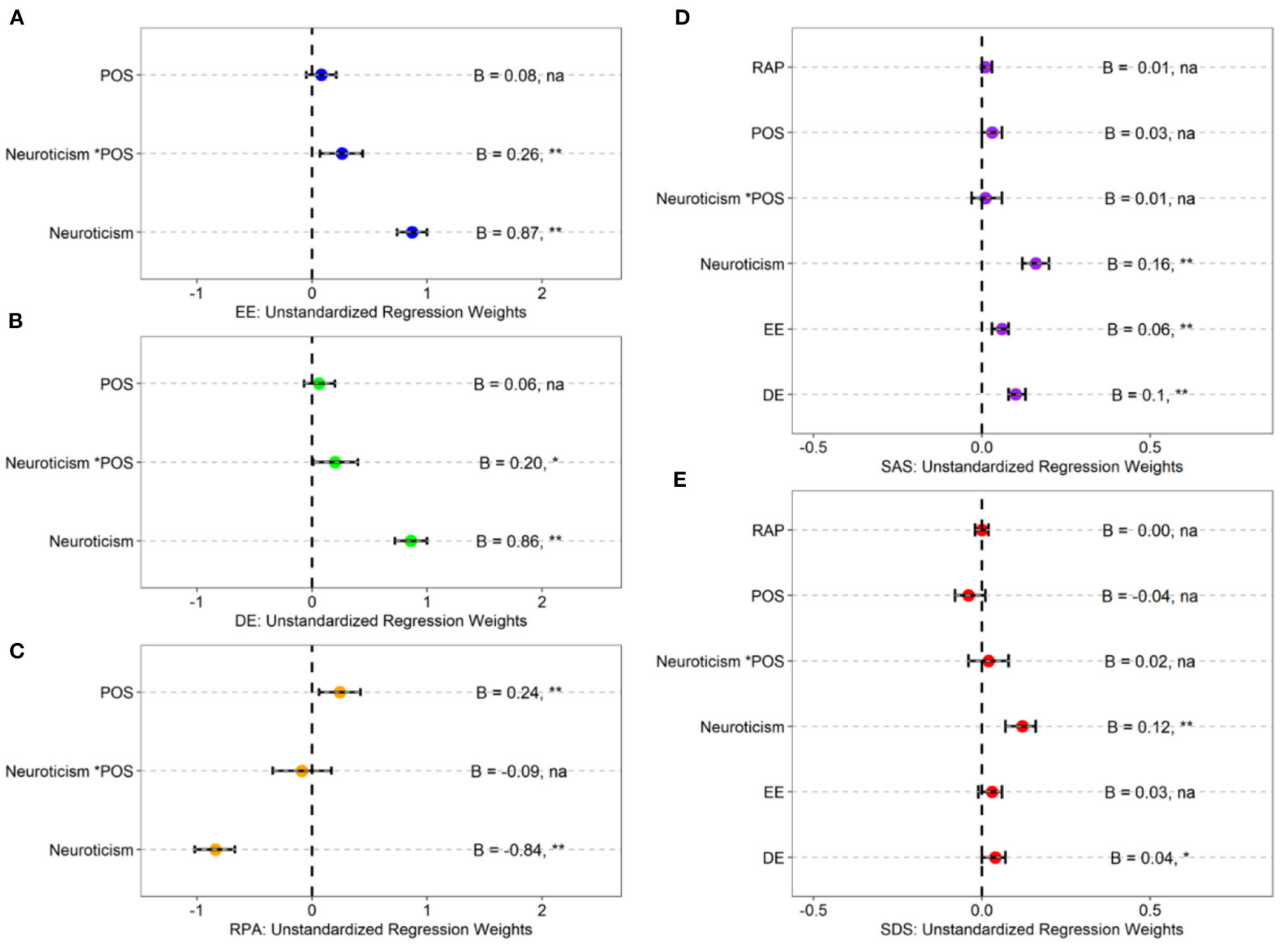
Unstandardized regression coefficients of the hypothesized model (*N* = 716). POS, perceived organizational support; EE, emotional exhaustion; DE, depersonalization; RPA, reduced personal accomplishment. **(A)** Indicates model 1. **(B)** Indicates model 2. **(C)** Indicates model 3. **(D)** Indicates model 4. **(E)** Indicates model 5. ^*^ indicates *p* < 0.05; ^**^ indicates *p* < 0.01; na indicates *p*> 0.05.

Corresponding with the multiple regression analysis (49) and following the recommendation by Aiken et al. (50), we mean-centered the two continuous variables (Neuroticism and POS) and tested the independent variable (Neuroticism), moderation variable (POS), and the interaction of independent and moderation variables (Neuroticism^*^POS) to predict mediation variables (EE, DE, RPA). The path analysis model was conducted, and overall fitness of the path model was acceptable {χ^2^*/df* = 41.03, *p* < 0.01; *RMSEA* = 0.24 [*CI* (0.20, 0.29)]; *CFI* = 0.95; *GFI* = 0.96; *SRMR* = 0.07}. Just as depicted in Tables 2, 3 illustrate the hypothetic model's unstandardized regression coefficients. Figure 1 shows the moderation results.

**Table 2:** Regression results using EE (Model 1), DE (Model 2), and RPA (Model 3) as the criterion.

**Model**	**Predictor**	***b***	***b*** **95% CI** **(LL, UL)**	***sr* ^2^**	***sr*^2^** **95% CI** **(LL, UL)**
Model 1	Neuroticism	0.87^**^	[0.74, 1.00]	0.19	[0.14, 0.24]
	POS	0.08	[−0.05, 0.21]	0.00	[–.00, 0.01]
	Neuroticism ^*^POS	0.26^**^	[0.07, 0.44]	0.01	[−0.00, 0.02]
Model 2	Neuroticism	0.86^**^	[0.72, 1.00]	0.18	[0.13, 0.23]
	POS	0.06	[−0.07, 0.20]	0.00	[−0.00, 0.00]
	Neuroticism ^*^POS	0.20*	[0.01, 0.40]	0.00	[−0.00, 0.01]
Model 3	Neuroticism	−0.84^**^	[−1.02, −0.67]	0.11	[0.06, 0.15]
	POS	0.24^**^	[0.06, 0.42]	0.01	[−0.00, 0.02]
	Neuroticism ^*^POS	−0.09	[−0.34, 0.17]	0.00	[−0.00, 0.00]

*b Represents unstandardized regression weights. sr^2^ represents the semi-partial correlation squared. LL and UL indicate the lower and upper limits of a confidence interval, respectively.*

* *Indicates p < 0.05.*

** *Indicates p < 0.01.*

*EE, emotional exhaustion; DE, depersonalization; RPA, reduced personal accomplishment; POS, perceived organizational support*.

**Table 3:** Regression results using SAS (Model 4) and SDS (Model 5) as the criterion.

	**Predictor**	***b***	***b*** **95% CI** **(LL, UL)**	***sr^**2**^***	***sr*^2^** **95% CI** **(LL, UL)**
Model 4	Neuroticism	0.16^**^	[0.12, 0.20]	0.06	[0.03, 0.08]
	POS	0.03	[−0.00, 0.06]	0.00	[−0.00, 0.01]
	Neuroticism^*^POS	0.01	[−0.03, 0.06]	0.00	[−0.00, 0.00]
	EE	0.06^**^	[0.03, 0.08]	0.01	[0.00, 0.03]
	DE	0.10^**^	[0.08, 0.13]	0.05	[0.03, 0.07]
	RPA	0.01	[−0.00, 0.03]	0.00	[−0.00, 0.01]
Model 5	Neuroticism	0.12^**^	[0.07, 0.16]	0.03	[0.00, 0.05]
	POS	−0.04	[−0.08, 0.01]	0.00	[−0.00, 0.01]
	Neuroticism^*^POS	0.02	[−0.04, 0.08]	0.00	[−0.00, 0.00]
	EE	0.03	[−0.01, 0.06]	0.00	[−0.00, 0.01]
	DE	0.04^*^	[0.00, 0.07]	0.01	[−0.00, 0.02]
	RPA	−0.00	[−0.02, 0.02]	0.00	[−0.00, 0.00]

*b Represents unstandardized regression weights. sr^2^ represents the semi-partial correlation squared. LL and UL indicate the lower and upper limits of a confidence interval, respectively.*

* *Indicates p < 0.05.*

** *Indicates p < 0.01.*

EE, emotional exhaustion; DE, depersonalization; RPA, reduced personal accomplishment; POS, perceived organizational support; SAS, anxiety; SDS, depression; Neuroticism^*^POS, the interaction between Neuroticism and POS.

As shown in Table 2 and Figure 1, neuroticism (EE: *B* = 0.87, *p* < 0.01; DE: *B* = 0.86, *p* < 0.01) and Neuroticism^*^POS (*B* = 0.26, *p* < 0.01; DE: *B* = 0.20, *p* < 0.01) were reliable predictors of EE (Model 1) and DE (Model 2). Neuroticism was a negative predictor about RPA (*B* = −0.84, *p* < 0.01), while POS was a positive predictor (*B* = 0.24, *p* < 0.01). The interaction of neuroticism and POS was not significant (*p* > 0.05).

**Figure 2:**
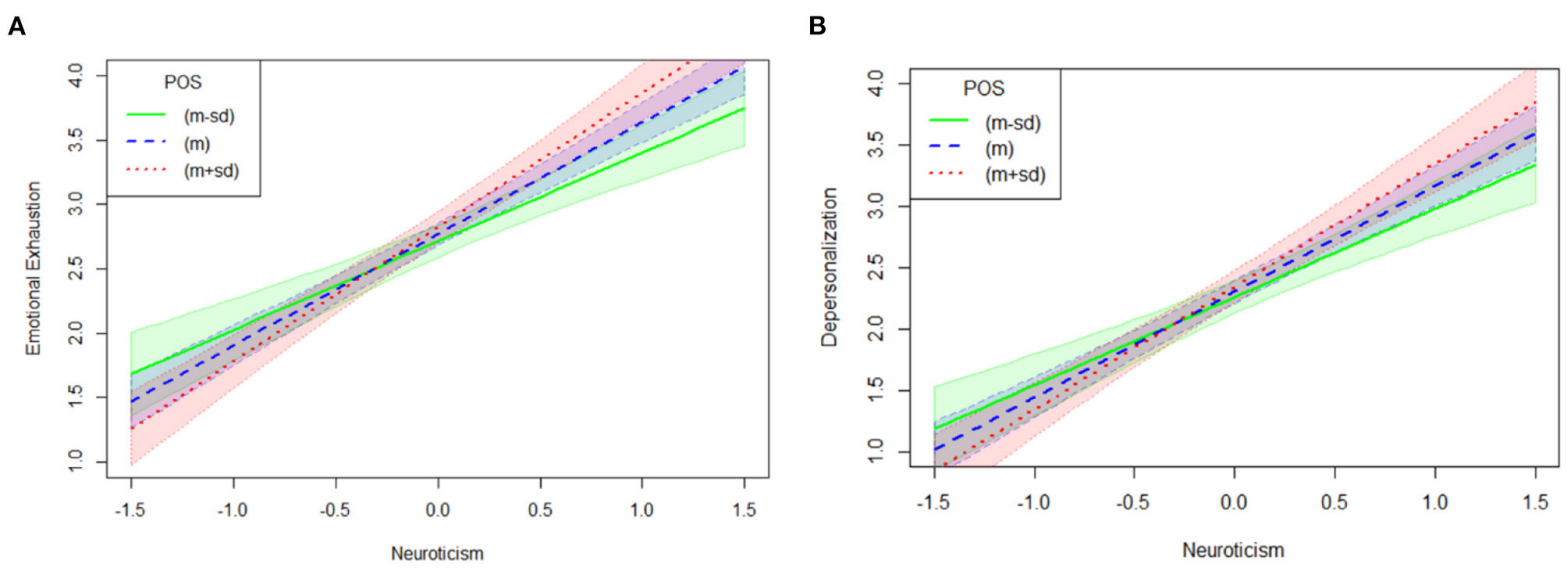
Interaction of neuroticism and POS on emotional exhaustion and depersonalization (*N* = 716). POS, perceived organizational support. **(A)** Indicates the interaction of neuroticism and POS on emotional exhaustion. **(B)** Shows the interaction of neuroticism and POS on depersonalization.

In terms with the statistical significance of the interaction between neuroticism and POS for emotional exhaustion and depersonalization, this result indicated that the association between neuroticism and emotional exhaustion increased in magnitude as the levels of POS increased from low [−1SD; β = 0.77, *SE* = 0.08, *p* < 0.001, 95% *CI* = (0.61, 0.92)] to moderate [Mean; β = 0.86, *SE* = 0.07, *p* < 0.001, 95% *CI* = (0.73, 0.99)] to high (+1SD; β = 0.99, *SE* = 0.08, *p* < 0.001, 95% *CI* = (0.84, 1.14)]. The positive relationship between neuroticism and emotional exhaustion was reinforced for the firefighters with higher levels of POS, which was an unexpected result (see Figure 2A).

The results also indicated that the association between neuroticism and depersonalization increased in magnitude as the levels of POS increased from low [−1SD; β = 0.78, *SE* = 0.08, *p* < 0.001, 95% *CI* = (0.62, 0.94)] to moderate [Mean; β = 0.86, *SE* = 0.07, *p* < 0.001, 95% *CI* = (0.72, 0.99)] to high [+1SD; β = 0.96, *SE* = 0.08, *p* < 0.001, 95% *CI* = (0.80, 1.12)]. The positive relationship between neuroticism and depersonalization was reinforced for the firefighters with higher levels of perceived organizational support, which was an unexpected result (see Figure 2B).

### Multiple Mediation Analysis

The next step was to evaluate the mediating role of job burnout in the relationship between neuroticism and mental health (see Table 4). Results revealed that neuroticism positively predicted EE and DE (*p* < 0.01) and negatively predicted RPA (*p* < 0.01). The direct effect of neuroticism on anxiety (*B* = 0.16, *p* < 0.01) and depression (*B* = 0.12, *p* < 0.01) were significant.

**Table 4:** Test of mediating effects.

**Paths**	**Unstandardized *B* (*SE*)**	**Unstandardized** **95%** ***CI***	
		**Low**	**High**
**Direct paths**			
Neuroticism—SAS	0.16^***^ (0.02)	0.12	0.20
Neuroticism—SDS	0.12^***^ (0.03)	0.07	0.16
**Indirect paths**			
Neuroticism—EE—SAS	0.05^***^ (0.01)	0.03	0.07
Neuroticism—DE—SAS	0.09^***^ (0.01)	0.06	0.12
Neuroticism—RPA—SAS	−0.01 (0.01)	−0.02	0.001
Neuroticism —EE—SDS	0.02 (0.02)	−0.01	0.06
Neuroticism—DE—SDS	0.03^**^ (0.02)	0.003	0.06
Neuroticism—RPA—SDS	0.00 (0.01)	−0.01	0.02

*EE, emotional exhaustion; DE, depersonalization; RPA, reduced personal accomplishment; SAS, anxiety; SDS, depression.*

** *Indicates p < 0.01.*

*** *Indicates p < 0.001*.

The indirect association between neuroticism and anxiety through EE [β = 0.05, *SE* = 0.01, *p* < 0.001, 95% *CI* = (0.03, 0.07)] and DE [β = 0.09, *SE* = 0.01, *p* < 0.001, 95% *CI* = (0.07, 0.11)], was statistically significant, respectively. Similarly, the indirect association between neuroticism and depression through DE [β = 0.03, *SE* = 0.01, *p* < 0.001, 95% *CI* = (0.01, 0.05)], was statistically significant, respectively. The indirect role of RPA for anxiety and depression was not significant (*p* > 0.05).

## Discussion

Our results suggested that firefighters with the neurotic trait are more prone to experience anxiety and depression, which is consistent with the general population (12, 51). The relationship among neuroticism, depression and anxiety has been widely discussed (11, 52). Neuroticism is widely defined as a long-term tendency to experience negative emotions, especially when a person is threatened, depressed, or experiencing loss. High level of neuroticism results in skeptical emotional disorders and maladapted social or interpersonal relationships.

Inconsistent with our expectations, POS positively regulates the relationship between neuroticism and job burnout. For neurotic firefighters, the more organizational support they perceive can result in a higher level of burnout, especially in the dimensions of emotional exhaustion and depersonalization. In China, firefighters are recruited from the army. Under such circumstances, once soldiers perceive that their organizations or commanders are concerned, they feel anxious instead of support. Supposedly, organizational structure and cultural differences lead to such a phenomenon. Therefore, perceived organizational support may have a reverse inhibitory effect on firefighters (53).

In line with the rich literature of previous studies, we found a strong correlation between burnout and anxiety, depressive symptoms. Interestingly, we found that depersonalization could mediate the relationship between neuroticism and both anxiety and depression. According to Melamed et al. (23), depersonalization is characterized by employees' tendency to regard others as objects. High level of depersonalization indicates that employees tend to be cynical, detached, or emotionally indifferent to colleagues and customers (i.e., rescuers and survivors) (54). Undertaking complex and heavy rescue tasks, firefighters experience many negative events frequently, which can easily lead to mental problems and emotional disorders.

Moreover, incorrectly understanding the will of organizations might worsen the situation. In sum, our study did well in researching the relationship between the neuroticism trait and mental health by considering the moderating role of POS and the mediating role of the three components of burnout among Chinese firefighters. Thereby current study can reveal some facts about the mental health condition of firefighters in China. Also, leaders or decision-makers can take action in terms of our models.

### Limitations and Future Directions

Some limitations in this study should be noted. First, our analysis does not infer causality between variables as in all the cross-sectional designs. Though we found the indirect association between neuroticism and anxiety through EE and DE was significant, DE played an indirect role through neuroticism to depression. In future research, network analysis (55) may be an excellent choice to explore the causality among main variables.

Second, we rely on the “subjective” measurement of anxiety and depression and occupational level variables. Some firefighters were mentally defensive in post-survey interviews though we repeatedly told them that this study was confidential and no results would be given to their senior commanders. This situation required us to use “objective” indicators (i.e., indicators that do not involve the perception or evaluation of respondents) to reduce the impact of various response deviations (such as social expectation deviation) (56).

## Conclusion

Reducing firefighters' job burnout can benefit their physical and mental health and our society. Hence, it is crucial to explore the mediating mechanisms of job burnout influencing mental health, especially considering the moderating role of perceived organizational support among firefighters.

Our findings demonstrated that neuroticism traits influenced anxiety and depression through job burnout, and the role of perceived organizational support moderated the effects of neuroticism on emotional exhaustion and depersonalization in the same direction. Future research should explore other contextual and individual mediators and moderators of the relationship between firefighters' personality traits and mental health to clarify the matter further.

## Data Availability Statement

The raw data supporting the conclusions of this article will be made available by the authors, without undue reservation.

## Ethics Statement

The studies involving human participants were reviewed and approved by School of Psychology, Nanjing Normal University. The patients/participants provided their written informed consent to participate in this study.

## Author Contributions

CS: study design. LZ and CL: data collection. YT: analysis, interpretation, and drafting of the manuscript. ZM, WH, and YZ: critical revision of the manuscript. All authors contributed to the article and approved the submitted version.

## Conflict of Interest

The authors declare that the research was conducted in the absence of any commercial or financial relationships that could be construed as a potential conflict of interest.

## Publisher's Note

All claims expressed in this article are solely those of the authors and do not necessarily represent those of their affiliated organizations, or those of the publisher, the editors and the reviewers. Any product that may be evaluated in this article, or claim that may be made by its manufacturer, is not guaranteed or endorsed by the publisher.
